# The Effects of Dairy Intake on Insulin Resistance: A Systematic Review and Meta-Analysis of Randomized Clinical Trials

**DOI:** 10.3390/nu11092237

**Published:** 2019-09-17

**Authors:** Kristen M. Sochol, Tanya S. Johns, Rupinder S. Buttar, Lovepreet Randhawa, Edeline Sanchez, Maya Gal, Katherine Lestrade, Massini Merzkani, Matthew K. Abramowitz, Yasmin Mossavar-Rahmani, Michal L. Melamed

**Affiliations:** Departments of Medicine and Epidemiology and Population Health, Albert Einstein College of Medicine, Bronx, NY 10461, USA; kristen.sochol@gmail.com (K.M.S.); tjohns@montefiore.org (T.S.J.); rubbybuttar@hotmail.com (R.S.B.); lvprt.randhawa90@gmail.com (L.R.); esanch01@syr.edu (E.S.); mayagal1707@gmail.com (M.G.); katherine.lestrade@gmail.com (K.L.); massini00@hotmail.com (M.M.); matthew.abramowitz@einstein.yu.edu (M.K.A.); yasmin.mossavar-rahmani@einstein.yu.edu (Y.M.-R.)

**Keywords:** dairy intake, insulin resistance, weight loss, waist circumference, diet, low-fat dairy

## Abstract

The incidence of type 2 diabetes mellitus (DM) has increased in the US over the last several years. The consumption of low-fat dairy foods has been linked with decreasing the risk of DM but studies have yet to show a clear correlation. We conducted a systematic review and meta-analysis of randomized clinical trials (RCTs) evaluating the effects of dairy intake on homeostatic model assessment of insulin resistance (HOMA-IR), waist circumference, and body weight. In MEDLINE and Embase, we identified and reviewed 49 relevant RCTs: 30 had appropriate data format for inclusion in the meta-analysis. Using the Review Manager 5 software, we calculated the pooled standardized mean differences comparing dairy dietary interventions to control for our outcomes of interest. For HOMA-IR (794 individuals), we found a mean difference of −1.21 (95% CI −1.74 to −0.67; *p*-value < 0.00001; *I*^2^ = 92%). For waist circumference (1348 individuals), the mean difference was −1.09 cm (95% CI 1.68 to −0.58; *p*-value < 0.00001; *I*^2^ = 94%). For body weight (2362 individuals), the dairy intake intervention group weighed 0.42 kg less than control (*p*-value < 0.00001; *I*^2^ = 92%). Our findings suggest that dairy intake, especially low-fat dairy products, has a beneficial effect on HOMA-IR, waist circumference, and body weight. This could impact dietary recommendations to reduce DM risk.

## 1. Introduction

There have been dramatic increases in the rates of obesity-associated diseases like type 2 diabetes mellitus (DM) potentially related to the modern lifestyle that is accompanied by excess food availability and reduced physical activity [1]. According to World Health Organization (WHO) 2016 report, the global prevalence of DM among adults over 18 years of age has risen from 4.7% in 1980 to 8.5% in 2014 [2]. In 2012, an estimated 1.5 million deaths were directly caused by diabetes mellitus [2]. Insulin resistance is a hallmark of type 2 DM and likely precedes clinical DM by many years. An excess intake of calories and the resulting increased body fat can lead to the development of insulin resistance and type 2 DM [3]. 

Modifying dietary composition would be an appealing alternative to drug-based therapies to prevent the development of insulin resistance and DM. This would greatly help the more than 1.1 billion people worldwide who are overweight and at risk of DM [4,5]. One such potential food component is low-fat dairy products. 

The data regarding the association of low-fat dairy products and body weight management is unclear. Animal and human in vitro studies consistently show an association of increased dairy intake with decreased lipogenesis and increased lipolysis, which could theoretically translate into body weight loss [6,7,8]. A recent meta-analysis by Geng et al. [9] suggested a beneficial effect of energy-restricted dairy consumption on body weight and composition. However, high dairy consumption in the absence of caloric restriction may be linked with increased body weight [9]. Observational studies have indicated a potential benefit of dairy products and their constituents on DM risk. Meta-analyses of cohort studies have demonstrated an inverse relationship between total dairy intake and type 2 DM risk [10,11]. A more recent large prospective cohort of ~63,000 Chinese men and women showed that daily intake of dairy products and daily milk consumption was associated with a modest decrease in DM risk [12]. In contrast, a Dutch prospective study on dairy product consumption showed no beneficial association with type 2 DM risk [13]. In the Framingham Offspring Study, a more complicated association between dairy intake and the risk of DM was found, with a protective association in participants with normal glycemia at baseline and only with certain dairy products [14]. In meta-analyses of observational studies on vitamin D and calcium intake (dairy constituents) in type 2 diabetes [15,16] there was a significantly lower incidence of type 2 DM in the highest dairy versus lowest dairy intake groups. However, in a meta-analysis of randomized clinical trials (RCTs), vitamin D supplementation had no effect on glycemic outcomes except in patients with baseline glucose intolerance [15,17]. While vitamin D is one component of dairy products for some dairy products such as milk in the US, but not necessarily overseas, there may be effects of metabolomic components as well as nutrients such as calcium and proteins that may affect glycemic status. Therefore, we conducted a systematic review and meta-analysis of RCTs testing the effects of dairy supplementation on insulin resistance assessed through homeostasis model assessment-insulin resistance (HOMA-IR), waist circumference, and body weight which are established risk factors for type 2 DM. 

## 2. Materials and Methods

### 2.1. Search Strategy

We conducted a search of MEDLINE and The Cochrane Library Central Register of Controlled Trials for RCTs from inception to July 2016 on the effect of dairy intake in adults related to insulin resistance or body mass index changes through weight loss. With the help of a medical librarian, we developed a search strategy using separate searches for the following medical subject headings: Dairy Product, in combination with each of the following Insulin Resistance, Weight Loss, Body Mass Index, Diet, and Reducing. In addition to Dairy Product, we also searched for individual components such as milk, butter, cheese, and yogurt. We limited the search to adults (≥18 years), RCTs, and the English language. Additionally, we retrieved more articles from reference lists of recovered articles, review articles, and personal reference lists. All disagreements for study selection, data abstraction, and study quality assessments were resolved through discussion between the authors.

### 2.2. Study Selection

Four independent authors (K.M.S., M.L.M., E.S., R.S.B.) reviewed each study and used criteria for inclusion/exclusion of studies established prior to the literature search to assess each citation by title and abstract, or by reading the publication. Studies had to fulfill the following criteria for eligibility: (a) Randomization should be used to allocate patients in groups; (b) intervention of dairy supplementation with or without an energy deficit or caloric restriction; and (c) reported at least one of the following outcomes of interest: homeostasis model assessment-insulin resistance (HOMA-IR), waist circumference or body weight collected at baseline and follow-up. We also directly contacted authors to obtain additional data from published manuscripts in order to analyze as many studies as possible. All data used in this analysis was in aggregate, de-identified form.

### 2.3. Data Abstraction

Five authors (K.M.S., E.S., M.M., R.S.B., and T.S.J.) independently abstracted relevant data using predesigned data abstraction tables. Data abstracted included (a) participant demographics; (b) treatment information (type of intervention, dose, and duration); (c) number of participants; (d) duration of follow-up; (e) outcome data of interest at baseline and follow-up; and (f) body mass index.

### 2.4. Study Quality and Risk of Bias Assessment

Two authors (R.S.B. and L.R.) independently reviewed all included studies and rated them on the basis of 5 criteria: (1) Intention-to-treat analysis, (2) blinding of the study, (3) reporting of adherence, (4) sample size and power calculations reported, and (5) completeness of cohort follow-up. Risk of bias (ROB) assessment for each study was performed using a Cochrane risk of bias assessment tool modified to the five criteria. When three or more of the quality criteria were rated as having a high or unclear risk, the overall ROB was rated as high. If two or less of the quality items were rated as having a high or unclear risk, the overall ROB was rated as low.

### 2.5. Publication Bias Assessment

To assess for publication bias for each outcome, we constructed Begg and Mazumdar’s funnel plots [18]. These funnel plots were drawn by using the standard error by standardized difference in means and confidence interval for all the studies included in our review.

### 2.6. Statistical Analyses

Standardized mean differences and their 95% confidence intervals (CI) were calculated using a random effects model as described by Dersimonian and Laird [19]. To assess heterogeneity across studies, we used the Cochran Q statistic based on the pooled standardized mean difference by Mantel–Haenszel, and measured inconsistency (*I*^2^; the percentage of total variance across studies attributable to heterogeneity rather than chance) of treatment effects across trials [20]. All *p* values were two-tailed and a *p*-value < 0.05 was considered statistically significant. All analyses were completed using Review Manager 5 (RevMan 5, Copenhagen, Denmark) computer software. 

### 2.7. Sensitivity Analyses

We also performed sensitivity analyses to ascertain the effect on the point estimates and heterogeneity statistics for all three of the outcomes. This included limiting our analyses to studies assessed to have a low risk of bias and excluding studies that had a physical activity component as part of the intervention. 

## 3. Results

### 3.1. Study Selection

**Figure 1:**
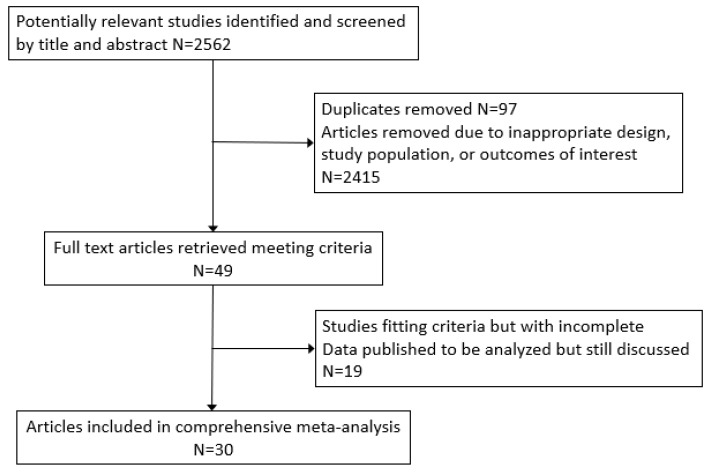
Flowchart of studies included in meta-analysis.

From the initial search and personal reference lists, there were 2562 articles to be screened by title and abstract, or full text if necessary. Ninety-seven duplicates were removed, and 2415 studies were removed by abstract and title due to inappropriate study design, study population, or outcomes of interest (Figure 1). Forty-nine full text articles were reviewed from the initial search. Several of these articles reported their findings in formats inappropriate for the meta-analysis and the authors were contacted. The authors who responded and provided data with the correct format were included in the meta-analysis (Table 1). The remaining studies were discussed but not included in the meta-analysis (Supplement Table S1) [21,22,23,24,25,26,27,28,29,30,31,32,33,34,35,36,37,38,39]. 

Thirty articles met inclusion criteria and were included in the meta-analysis [40,41,42,43,44,45,46,47,48,49,50,51,52,53,54,55,56,57,58,59,60,61,62,63,64,65,66,67,68,69] as seen in Table 1. The total sample size was 2900 with >50% female participants, but the distribution varied greatly across different studies. The mean age of subjects ranged from 18–63 years. Baseline study participant characteristics, intervention type, dose, and duration, along with specific study findings are shown in Table 1. The intervention descriptions and control group are outlined in Table 2. The following control groups were identified: 1) no intervention (usual diet), 2) a diet similar in structure and energy intake with a habitual or low dose of dairy products (<2 dairy products per day) or without the use of dairy, 3) placebo capsules, 4) an energy deficit diet, or 5) soy intake of a comparable energy intake. All except two studies tested the effects of high vs. low intakes of dairy [45,47]. Bowen et al. replaced the dairy products in controls with rice milk and soya while Benatar et al. eliminated dairy in controls with no substitution. A few of the studies also had a physical activity component as part of the overall intervention (Table 2) [42,48,52,53,56,62,63,68]. 

### 3.2. Dairy Intake and HOMA-IR

**Figure 2:**
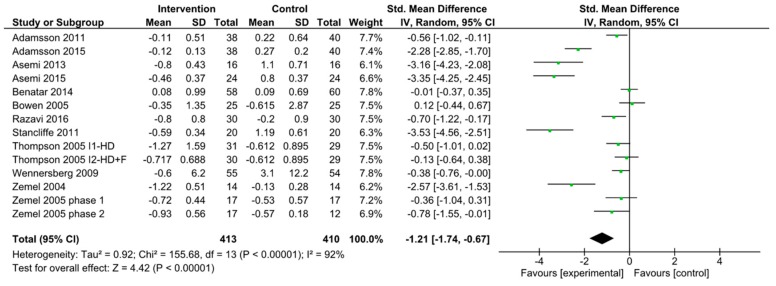
Forest plot of randomized clinical trials of dairy intake and HOMA-IR. Abbreviations: F, fiber; HD, high dairy diet.

**Figure 3:**
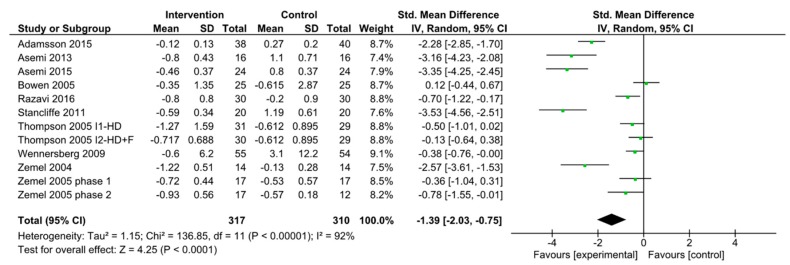
Forest plot of randomized clinical trials of dairy intake and HOMA-IR with BMI >25. Abbreviations: F, fiber; HD, high dairy diet.

When combining all studies of dairy intake reporting HOMA-IR, there were fourteen interventions studied from twelve articles [40,41,43,44,45,47,57,60,63,64,65,66]. Of these, one study had two intervention arms tested against the same control [63]. For the meta-analysis, we used the same control data for both intervention arms. A study comparing a high dairy diet to a low dairy diet conducted two separate randomized trials: One trial included 34 patients with caloric maintenance (phase 1) and the other included 29 patients who were under caloric restriction (phase 2) [66]. Separate meta-analysis was performed for each phase. The pooled standardized difference in means between the dairy and placebo groups in HOMA-IR was −1.21(95% CI −1.74 to −0.67; *p*-value < 0.00001; *I*^2^ = 92%) (Figure 2). Increase in dairy intake was also found to be associated with improved oxidative stress markers and HOMA- IR [43,60]. Within the fourteen treatment interventions of dairy and HOMA-IR, eleven of the interventions included only subjects with BMI >25 kg/m^2^ [41,43,44,47,57,60,63,64,65,66]. We combined these studies and the pooled standardized difference in means was −1.39 (95% CI −2.03 to −0.75; *p*-value < 0.00001; *I*^2^ = 92%) (Figure 3). This is a slightly greater reduction in HOMA-IR compared to when including all participants from all studies.

### 3.3. Dairy Intake and Waist Circumference

**Figure 4:**
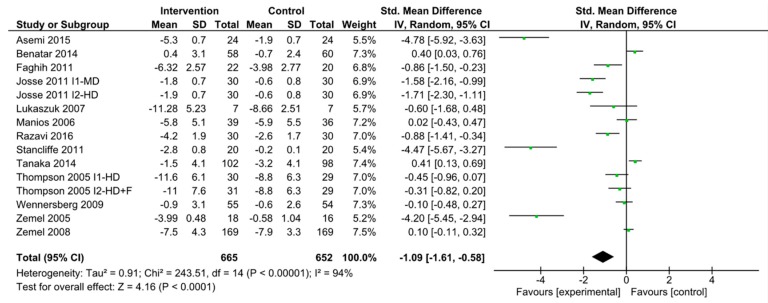
Forest plot of randomized clinical trials of dairy intake and waist circumference. Waist circumference change measured in cm. Abbreviations: F, fiber; HD, high dairy diet; MD, moderate dairy diet.

Because changes in abdominal adiposity impacts insulin resistance, we examined whether dairy intake may affect changes in waist circumference [70,71]. We found 13 studies with 17 interventions comparing the effect of dairy on waist circumference [44,45,49,53,55,56,57,60,61,63,64,66,68]. Of these, 2 studies [45,55] had controls with no dairy while the other studies did not completely exclude dairy from the control diet. Two studies had two intervention arms tested against the same control for which the meta-analysis was performed separately [53,63]. The pooled standardized difference in means was −1.09 cm (95% CI −1.61 to −0.58; *p*-value < 0.00001; *I*^2^ = 94%) comparing high diary intake to the control group (Figure 4). 

### 3.4. Dairy Intake and Body Weight 

**Figure 5:**
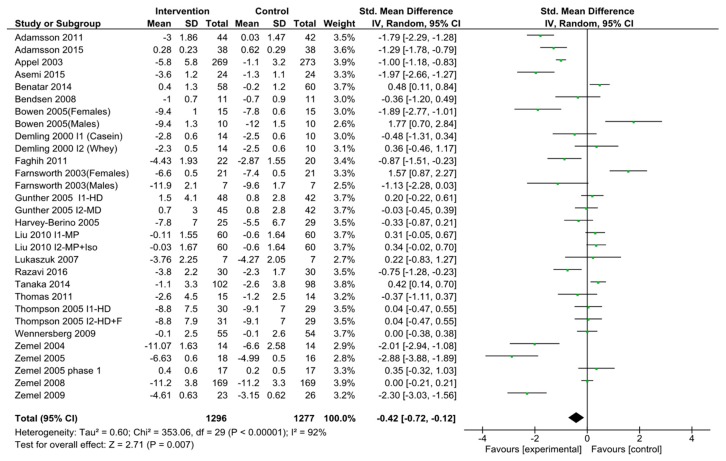
Forest plot of randomized clinical trials of dairy intake and body weight. Body weight change measured in Kg. Abbreviations: F, fiber; Iso, isoflavones; HD, high dairy diet; MD, moderate dairy diet; MP, milk protein.

We found 25 studies with 30 intervention arms [40,41,42,44,45,46,47,48,49,50,51,52,54,55,57,61,62,63,64,66,68,69,72,73]. Out of 25 studies, 4 studies had multiple intervention arms. For these studies, each intervention arm was treated as a separate trial [48,51,54,63]. Further, two more studies provided data for male and female participants separately, and were included in meta-analysis accordingly [47,50]. Another study was conducted in two phases but, accurate body weight change data was available for only the first phase. [66] Hence, the second phase was not included in the analysis. The pooled standardized difference in means was −0.42 kg (95% CI −0.72 to −0.12; *p*-value < 0.00001; *I*^2^ = 92%) comparing high dairy intake to the control group (Figure 5).

### 3.5. Risk of Bias

**Figure 6:**
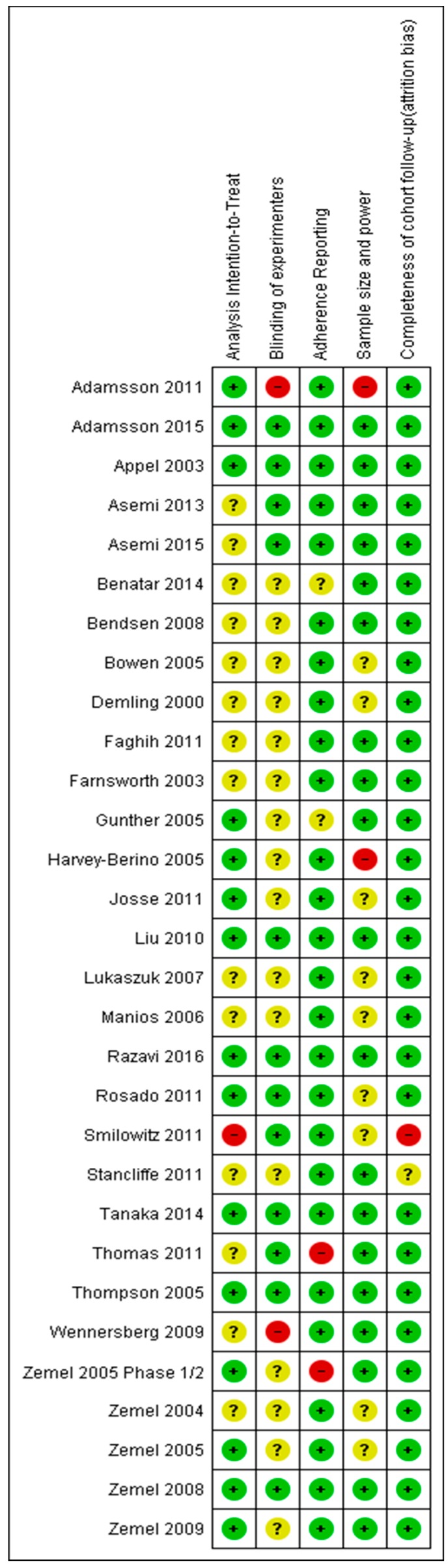
Risk of bias summary for all studies in the meta-analysis. Green= Low risk of bias, Yellow= Unclear risk, Red= High risk of bias.

**Figure 7:**
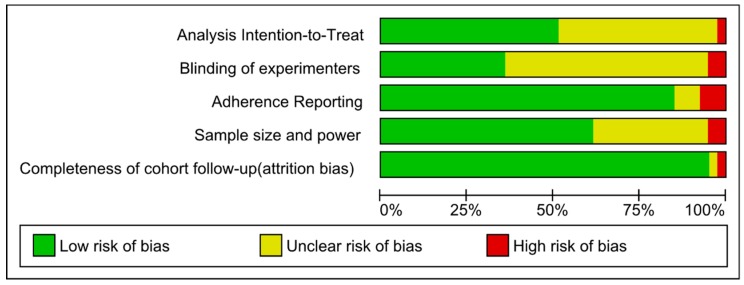
Risk of bias graph as percentages across all included studies.

The ROB assessment for each study is shown Figure 6. A total of 26% of studies were assessed to have high risk of bias. For the vast majority studies, it was unclear if the investigators were blinded to study arms (Figure 7). The second most common cause of bias or unclear bias was whether the studies used intention-to-treat analysis. More than 90% of studies reported on completeness of follow-up/ attrition. 

### 3.6. Publication Bias

The funnel plots for all three outcomes did not suggest significant publication bias (Supplemental Figures S1–S3). 

### 3.7. Sensitivity Analyses

Limiting our analysis to studies that were assessed to have low risk of bias did not significantly change the point estimates or heterogeneity statistics (Q or *I*^2^) for all three outcomes (Supplemental Figures S4–S6). Similarly excluding studies with a physical activity component did not significantly alter point estimates or heterogeneity statistics for all three outcomes (Supplemental Figures S7–S9).

## 4. Discussion

Our findings suggest that a diet which includes low-fat dairy products will decrease HOMA-IR in individuals, and therefore potentially decrease the risk of insulin resistance and type 2 DM. We also found a significantly lower waist circumference as well as body weight in individuals randomized to high dairy intake, suggesting that effects on HOMA-IR may be secondary to changes in body composition. We also found significant heterogeneity in the results which did not go away when restricting the analysis to only studies with low risk of bias or studies without a physical activity component as part of the intervention. The trials included have varying dietary dairy interventions, comparison groups (controls), and study populations which all likely contribute to the observed heterogeneity.

Our findings support previous observational studies that have shown an inverse association between diary intake and type 2 DM risk [10,11]. HOMA-IR is a reliable marker of insulin resistance [74]. Lower HOMA-IR levels have been linked to lower risks of several diseases including type 2 DM, cardiovascular, cerebrovascular, and peripheral artery disease in large-scale prospective and cross-sectional studies such as the National Health and Nutrition Examination Survey (NHANES) [75]. In addition to HOMA-IR, waist circumference is another measure used to estimate the risk of type 2 DM [73]. Increased waist circumference appears to be an important contributor to the metabolic syndrome, which is a cluster of abnormalities including insulin resistance, dyslipidemia, and high blood pressure. Moreover, it more accurately reflects central obesity and visceral fat which are closely linked to the pathophysiology of insulin resistance [76].

There are several possible mechanisms to explain the proposed effects of low-fat dairy on insulin resistance. Calcium, vitamin D, casein, and whey proteins are all present in low-fat fortified dairy products and have been independently described as potential regulators of body fat, waist circumference, and insulin resistance. Zemel et al. have proposed a mechanism describing the relationship between low-calcium intake and body fat accumulation. Increases in body fat are associated with increased risk of insulin resistance [3]. Zemel et al. have also shown that decreases in extracellular calcium levels due to decreased calcium intake followed by a subsequent rise in calcitriol result in an increase in intracellular calcium levels. This, in turn, seems to promote energy storage in human adipocytes by stimulating the expression and activity of fatty acid synthase and by inhibiting lipolysis [77]. The calcium response sequence is located at the fatty acid synthase promoter region on the human genome. Increasing intracellular calcium has been shown to stimulate the expression and activity of fatty acid synthase. The increased expression of fatty acid synthase is coordinated with inhibition of lipolysis by the increased intracellular calcium levels [78,79]. Another possible mechanism through which calcium regulates body fat is that increased calcium intake has been shown to promote fat cell apoptosis. This apoptotic effect is through inhibition of UCP2 expression, a regulator of apoptosis [80]. Additionally, high intakes of calcium through supplements or dairy intake have been associated with diminished fat absorption in the gut because insoluble calcium fatty acid soaps or calcium bound to bile acids will increase fecal fat excretion [81,82].

Vitamin D is another constituent of dairy with several possible effects on insulin resistance through pancreatic beta-cell function, insulin sensitivity, and inflammation. Vitamin D has an effect on the insulin response to glucose stimulation [83]. This may be due to the fact that the circulating active form of vitamin D, 1, 25-dihydroxyvitamin D_,_ binds directly to the beta-cell vitamin D receptor. In addition, activation of vitamin D may occur within the beta-cell by the 1-alpha-hydroxylase enzyme, which has been shown to be expressed in beta-cells [84], and this could explain the effect on insulin release. Vitamin D might directly influence insulin action by stimulating the expression of the insulin receptor on monocytes and thereby enhancing insulin responsiveness for glucose transport [85,86,87,88]. However, in a meta-analysis by Sarathy et al. on short-term vitamin D supplementation on glucose metabolism in end-stage kidney disease was found to be associated with lower fasting glucose levels with no change in fasting insulin levels [89]. In a recent intervention study of dairy intake vs. soy intake, dairy was shown to suppress the oxidative and inflammatory stress associated with overweight and obese subjects [39]. Vitamin D alone or as a component of dairy has been linked to the production and effects of cytokines and therefore may help to decrease insulin resistance [90,91,92,93].

Proteins in dairy foods such as casein and whey have been shown to have effects in reducing insulin resistance, hypertension, arterial stiffness, and LDL cholesterol in both human and animal studies [94,95,96]. In a double-blind, randomized, and controlled cross-over clinical trial of twelve subjects with type 2 DM taken off their hypoglycemic therapy and studied after consuming medium calorie high protein mixed meals containing whey protein, casein, or a free amino acid mixture; the ingestion of casein and whey resulted in significantly higher beta-cell secretion and postprandial aminoacidemia than the ingestion of the free amino acid mixture [97]. In patients with type 2 DM, progressive deterioration in beta-cell function and mass are well established phenomena. Chronic stimulation of beta-cells, through postprandial aminoacidemia, may promote beta-cell proliferation and limit progressive deterioration. Postprandial aminoacidemia also has a stimulatory effect on the amplitude and possibly duration of muscle protein synthesis. The preservation of muscle can be beneficial in type 2 DM to prevent declines in metabolic rate, which lead to fat mass accumulation and obesity [98,99]. A study by Demling et al. demonstrated that casein protein supplementation in weight training by individuals on a calorie-restricted diet leads to accelerated fat loss and lean mass gain [48]. These changes have been found to be associated with decreased insulin resistance.

Dairy intake may also promote weight loss and decrease insulin resistance by inducing satiation, thereby limiting intake of excess calories. In a meta-analysis of clinical trials by Onvani et al., the consumption of more than 500 mL of dairy products was significantly associated with increased satiety and decreased energy intake in the following meal [100].

There are limitations to the current analysis which should be mentioned. While BMI is often an important measure of risk for insulin resistance and type 2 DM, not enough of the studies reported adequate data on BMI to be included in the meta-analysis. However, results from NHANES III analysis comparing trends in BMI and waist circumference suggest that the harmful health consequences associated with obesity may be increasingly underestimated by trends in BMI alone and that waist circumference is an important independent risk factor to assess [101]. Another limitation of our study is that in evaluating food group (i.e., diary) we cannot discern the effects of individual foods (such as cheese, milk, yogurt) or components (such as calcium, vitamin D, or whey/ casein protein intake). This report was also limited because the many of the intervention studies examining the effects of dairy reported HOMA-IR insufficiently for meta-analysis. Furthermore, we did not have information on baseline glycemic status for the individuals included in the studies, which has been shown to influence the relationship between dairy intake and prediabetes or type 2 DM risk in the adults [14]. There was also significant heterogeneity among the dairy intervention trials included in our meta-analyses. Nonetheless, these findings provide valuable insight for healthcare providers as well as the general public on the possible positive impact of incorporating low-fat dairy into a healthy lifestyle. 

## 5. Conclusions

Our findings suggest that dairy intake, especially low-fat dairy products, has a beneficial effect on HOMA-IR, waist circumference, and body weight. Some or all the mechanisms discussed above that are attributable to the components of low-fat dairy may work synergistically to improve insulin sensitivity, making it an appealing possible option for lowering the risk of insulin resistance and type 2 DM. Low-fat dairy products are easy to find and affordable in comparison to over the counter or prescription supplements. For a more definitive answer, larger scale randomized controlled trials of low-fat dairy intake and its impact on insulin resistance must be performed, especially in overweight and obese populations.

## Figures and Tables

**Table 1:** Randomized controlled trials of dairy intake included in the meta-analysis.

Study Author, Year	Sample Size	Sex	Age Mean ± SD or Range (Years)	BMI Mean ± SD or Range (kg/m^2^)	Country of Origin	Study Duration (Weeks)	Findings Intervention vs. Control
Adamsson, 2011 [40]	I = 44 C = 42	37% M 63% F	I = 52.6 ± 7.8 C = 53.4 ± 8.1	I = 26.3±3.3 C = 26.5 ± 3.3	Sweden	6	↓ Body weight ↓ BMI ↓ HOMA-IR
Adamsson, 2015 [41]	I = 38 C = 40	40% M 60% F	I = 54.9 ± 7.8 C = 54.4 ± 9	I = 28.2 ± 2.5 C = 28.5 ± 2.4	Sweden	12	↔ HOMA -IR ↔ Body weight
Appel, 2003 [42]	I = 269 C = 273	38% M 62% F	50 ± 8.9	18.5–45	United States	26	↓Body weight
Asemi, 2013 [43]	I = 16 C = 16	100% F	I = 27.7 ± 5.4 C = 29.7 ± 5.6	I = 30.2 ± 4.6 C = 29.7 ± 3.3	Iran	4	↓ Insulin ↓ FPG ↓ HOMA-IR ↔ BMI ↔ Body weight
Asemi, 2015 [44]	I = 24 C = 24	100% F	I = 29.4 ± 6.2 C = 30.7 ± 6.7	I = 31.5 ± 5.7 C = 29.1 ± 3.2	Iran	8	↓ HOMA-IR ↓ WC ↓ Body weight ↓ BMI
Benatar, 2014 [45]	I = 60 C = 60	36% M 64% F	I = 46.3 ± 10.5 C = 48.6 ± 12	I = 24.6 ± 4.1 C = 24.3 ± 4.0	New Zealand	4	↔ WC ↔ Body weight ↔ Insulin ↔ HOMA-IR
Bendsen*, 2008 [46]	I = 11 C = 11	45% M 55% F	18–50	25–31	Denmark	3	↔ Body weight
Bowen, 2005 [47]	I = 25 C = 25	60% M 40% F	25–64	25–35	Australia	16	↔ Body weight ↔ HOMA-IR
Demling, 2000 [48]	I1 = 14 I2 = 14 C = 10	100% M	28–40	29 ± 4.1	United States	12	↔ Body Weight ↓ %Body fat ↑ %Lean body mass
Faghih, 2011 [49]	I = 22 C = 20	100% F	20–50	25–40	Iran	8	↓ Body weight ↓ BMI ↓ WC
Farnsworth, 2003 [50]	I = 28 C = 29	25% M 75% F	I = 51.2 ± 3.1 C = 49.6 ± 2.7	I = 34.1 ± 1.0 C = 34.0 ± 1.1	Australia	16	↔ HOMA-IR ↔ Abdominal fat ↔ Body weight ↔ Fat mass ↑ Lean mass
Gunther, 2005 [51]	I1 = 48 I2 = 45 C = 42	100% F	I1 = 20.1 ± 2.5 I2 = 20.2 ± 2.4 C = 20.1 ± 2.4	I1 = 22.4 ± 2.6 I2 = 23.3 ± 3.9 C = 22.1 ± 3.1	United States	52	↔ Body weight ↔ BMI ↔ Body fat mass
Harvey-Berino, 2005 [52]	I = 25 C = 29	8% M 92% F	I = 45.2 ± 7.0 C = 45.1 ± 6.5	25–34.9	United States	52	↔ Body weight ↔ %Body fat ↔ Body fat
Josse, 2011 [53]	I1 = 30 I2 = 30 C = 30	100% F	I1 = 30 ± 1.0 I2 = 26 ± 1.0 C = 28 ± 1.0	I1 = 31.4 ± 0.6 I2 =31.8 ± 0.6 C = 31.5 ± 0.6	Canada	16	↓ Body fat ↓ Body weight
Liu, 2010 [54]	I1 = 60 I2 = 60 C = 60	100% F	I1 = 55.9 ± 3.8 I2 = 56 ± 4.4 C = 56.4 ± 4.7	I1 = 24.6 ± 3.4 I2 = 24.8 ± 3.8 C = 24.1 ± 3.8	China	26	↔ HOMA-IR ↔ Body weight
Lukaszuk, 2007 [55]	I = 7 C = 7	100% F	18-45	I = 33.9 ± 10.5 C = 38.4 ± 10.0	United States	8	↔ Body weight ↔ WC
Manios, 2006 [56]	I = 39 C = 36	100% F	I = 60.5 ± 0.7 C = 61.4 ± 0.8	I = 28.3 ± 0.6 C = 29.8 ± 0.9	Greece	22	↓ BMI
Razavi Zade, 2016 [57]	I = 30 C = 30	50% M 50% F	C = 42.8 ± 10.6 I = 39.7 ± 7.3	C = 28.3 ± 3.3 I = 28.5 ± 3.2	Iran	8	↓ Body weight ↓ BMI ↓ HOMA-IR
Rosado, 2011 [58]	I = 43 C = 41	100% F	I = 34.9 ± 5.6 C = 34.1 ± 5.9	I = 34.8 ± 3.4 C = 24.8 ± 3.8	Mexico	16	↔ WC ↔ Body weight ↔ BMI ↔ %Body fat ↔ Body mass
Smilowitz, 2011 [59]	I = 22 C = 23	20% M 80% F	I = 25.1 ± 5.3 C = 24.2 ± 4.7	I = 28.3 ± 3.0 C = 28.8 ± 2.7	United States	12	↔ Body weight ↔ % Body fat ↔ % Lean mass ↔ WC
Stancliffe, 2011 [60]	I = 20 C = 20	50% M 50% F	I = 34.4 ± 9.4 C = 39.5 ± 10.2	I = 30.1 ± 4.4 C = 31.2 ± 5.4	United States	12	↔ Body weight ↓ %Body fat ↓ WC ↓ HOMA-IR ↓ Insulin level
Tanaka, 2014 [61]	I = 102 C = 98	100% M	I = 41.7 ± 7.5 C = 41.7 ± 7.1	I = 27.2 ± 3.9 C = 26.8 ± 2.9	Japan	24	↔ Body weight ↔ %Body fat ↔ WC
Thomas, 2011 [62]	I = 15 C=14	100% F	29–45	I = 29.4 ± 2.0 C = 28.7 ± 2.2	United States	16	↔ Body weight ↔ WC ↔ %Body fat
Thompson, 2005 [63]	I1 = 30 I2 = 31 C = 29	14% M 86% F	25–70	30-40	United States	48	↔ Body weight ↔ Body fat ↔ WC ↓ HOMA-IR
Wennersberg, 2009 [64]	I = 55 C = 54	33% M 67% F	M = 51.2 ± 8.1 F = 56 ± 7.4	I = 30.1 ± 3.6 C = 30.0 ± 3.3	Norway	26	↔ Body weight ↔ BMI ↔ % Body fat ↔ WC ↓ HOMA-IR
Zemel, 2004 [65]	I = 14 C = 14	17% M 83% F	46 ± 8	30–39.9	United States	24	↓ Body weight ↓ Body fat ↓ Insulin
Zemel, 2005 [66] (Phase 1)	I = 17 C = 17	32% M 68% F	I = 42.5 ± 2.6 C = 41.3 ± 2.7	I = 34.1 ± 0.7 C = 34.9 ± 0.8	United States	24	↔ Body weight ↓ Body fat ↑ Lean mass ↓ WC ↓ Insulin
Zemel, 2005 [66] (Phase 2)	I = 17 C = 12	14% M 86% F	I = 41.7 ± 2.9 C = 41.7 ± 2.7	I = 35.6 ± 0.7 C = 35.4 ± 0.9	United States	24	↓ Body weight ↓ Body fat ↑ Lean mass ↓ WC ↓ Insulin
Zemel, 2005 [67]	I = 18 C = 16	29% M 71% F	I = 39 ± 10 C = 42 ± 6	I = 32.1 ± 0.4 C = 33.2 ± 0.9	United States	12	↓ Body fat ↓ WC
Zemel, 2008 [68]	I = 169 C = 169	**	I = 39.9 ± 7.2 C = 40.7 ± 6.8	I = 34.4 ± 3.2 C = 34.6 ± 3.1	United States	39	↔ Body weight ↔ BMI ↔ WC
Zemel, 2009 [69]	I = 32 C = 38	23% M 77% F	I = 25.5 ± 5.0 C = 25.3 ± 4.9	I = 28.8 ± 2.8 C = 29.3 ± 2.8	United States	12	↔ Body weight ↔ Lean mass ↓ WC

* Cross-over design; ** Data missing on sex; ↓ = decrease; ↑ = increase; ↔ = no difference. Abbreviations: BMI, body mass index; C, control; F, female; HOMA-IR, homeostatic model assessment of insulin resistance; I, intervention; M, male; SD, standard deviation; WC, waist circumference.

**Table 2:** Description of intervention and control diet for studies in the meta-analysis.

Study Author, Date	Dietary Intervention	Physical Activity Component
Adamsson, 2011 [40]	I = Nordic diet (low-fat dairy, fruit, berries, legumes, LDL-C lowering foods) C = Habitual diet with usual physical activity	No
Adamsson, 2015 [41]	I = Breakfast consisting of oat bran, LFM, low-fat yogurt, jam, raisins, fruits and berries, whole grain bread, low-fat spread, turkey meat, pickled herring, or mackerel C = Habitual diet with recommendation for Nordic foods.	No
Appel, 2003 [42]	I = Diet rich in low-fat dairy, fruit, vegetables, whole grains ((2–3 servings/day of low-fat dairy), DASH diet: calcium ~1250 mg/day C = Habitual diet. Advised once about effect of lifestyle (exercise, and DASH diet) on blood pressure	Yes
Asemi, 2013 [43]	I = Diet rich in low-fat dairy, fruit, vegetables, whole grains (DASH diet) in proportions similar to control diet: calcium ~1752 mg/day C = Recommended proportions of fat, protein, and carbs ~ 1082 mg calcium/day	No
Asemi, 2015 [44]	I = Diet high in low-fat dairy (3 servings/day), fruits, lean meat, and vegetables. (DASH diet: carb 52%, protein18%, total fat 30% with BMI based calories restriction in both groups, calcium ~1714 mg/day C = Diet high in grains, simple sugars, dairy (2 servings daily), calcium ~1037 mg/day	No
Benatar, 2014 [45]	I= High fat milk and dairy (extra 2–3 servings of dairy; ~ 1300 mg calcium) C = Dairy/day replaced with rice milk or soya	No
Bendsen, 2008 [46]	I = High dairy diet from low-fat dairy products (calcium 2300 mg/day) C = Low dairy diet (calcium 700 mg/day)	No
Bowen, 2005 [47]	I = High dairy intake from low-fat dairy products (calcium 2400 mg/day) C = Low dairy intake (calcium 500 mg/day)	No
Demling, 2000 [48]	I1 = 75 gm casein hydrolysate plus control diet I2 = 75 gm whey hydrolysate plus control diet C = non lipogenic, hypocaloric diet alone (80% of predicted needs).	Yes
Faghih, 2011 [49]	I = High dairy intake with 3 services of low-fat milk daily (3 servings per day; calcium) with 1300 mg/day of calcium; 500 calorie energy deficit C = Low dairy intake with 500–600 mg/day calcium; 500 calorie energy deficit	No
Farnsworth, 2003 [50]	I = High dairy protein intake from yogurt (200 gm), LFM (30 gm), or low-fat cheese (60 gm): ~2000–2400 mg calcium/day C= Low protein with no milk or limited milk products: 500 mg calcium/day	No
Gunther, 2005 [51]	I1 = Substitute with dairy as source for 1000–1100 mg calcium/day I2 = Substitute with dairy as source for 1300–1400 mg calcium/day C= Maintain current dietary consumption of calcium (~700 mg/day) (isocaloric diet for all groups)	No
Harvey-Berino, 2005 [52]	I = 3–4 servings dairy/day (equivalent to 1200–1400 mg calcium) C = 1 serving dairy/day (equivalent to 500 mg calcium) Dairy sources were milk cheese and yogurt	Yes
Josse, 2011 [53]	I1 = 3–4 servings of dairy/day (~950 mg calcium/day) I2 = 6–7 serving of dairy/day (~1650 mg calcium/day)) C = 0–1 serving dairy/day	Yes
Liu, 2010 [54]	I1 = 15 g milk protein on a daily basis in addition to usual diet I2 = 15 g milk protein and 100 mg isoflavones in addition to usual diet C = 15 g soy protein and 100 mg isoflavones in addition to usual diet	No
Lukaszuk, 2007 [55]	I = 720 mL skimmed milk/day (~900 mg calcium/day) C = 720 mL soy milk/day (~1350 mg calcium/day)	No
Manios, 2006 [56]	I = Low-fat dairy products (milk and yogurt) fortified with calcium and vitamin D (~1250 mg calcium/day) C = Regular diet (~730 mg calcium/day)	Yes
Razavi Zade, 2016 [57]	I = Energy restricted DASH diet consisted of 52–55% carbohydrates, 16–18% proteins and 30% total fats (~1800 mg calcium/day). The DASH diet was rich in fruits, vegetables, whole grains, and low-fat dairy products; and low in saturated fats, cholesterol, refined grains, and sweets C = Energy restricted regular diet with 52–55% carbohydrates, 16–18% protein and 30% total fats (~1064 mg calcium/day).	No
Rosado, 2011 [58]	I = 750 mL of low-fat milk/ day in addition to an energy-restricted diet of –500 kcal/day (~1000 calcium/day) C = Energy-restricted diet of −500 kcal/day with no intake of milk	No
Smilowitz, 2011 [59]	I = High dairy diet (placebo-supplemented) providing a 2093 kJ/day deficit (500 kcal/day) and containing 3 daily servings dairy products (milk, cheese, and/or yogurt) to bring the total calcium intake to 1400 mg/day C = 0–1 serving of dairy products/day, 500 mg calcium/day, and a daily placebo supplement. Control diet providing a deficit (500 kcal/day)	No
Stancliffe, 2011 [60]	I = Adequate dairy diet (3.5 servings dairy daily (~1200 mg calcium/day) C = low-dairy diet (<0.5 serving/day ~ less than 600 mg calcium/day)	No
Tanaka, 2014 [61]	I = Milk and dairy products (400 g/day). C = Regular diet	No
Thomas, 2011 [62]	I = 6-oz serving of fat-free yogurt (Yoplait Light Thick and Creamy ~200 mg calcium) containing 418 kJ 20 min before and immediately after each exercise session C = 6-oz serving of an isoenergetic placebo beverage containing 25 g of carbohydrate, 0 g of fat, and 0 g of protein	Yes
Thompson, 2005 [63]	I1 = 4 servings/day dairy (~1387 mg calcium/day) I2 = 4 servings/day dairy (~1439 mg calcium/day) with an increased amount of fiber (through additional whole grains, fruits, and vegetables) and with a reduction in glycemic index (foods with a glycemic index >100 were strongly discouraged). C = 2 servings/day dairy (~800 mg calcium/day) with 30% fat, 20% protein, and 50% carbohydrate. The diet was designed to provide an average level of calcium and fiber. Energy deficit of 500 calories	Yes
Wennersberg, 2009 [36]	I = 3 to 5 serving of dairy products in their diet daily including milk containing 0.5–3% fat (one portion defined as 200 g milk), yogurt or sour milk (1.0–5.4% fat, 200–250 g), cream or creme fraiche (12–40% fat, = 75 g), cheese (15–30%, 15–40 g), butter or butter containing spreads (40–80% fat, 3–10 g), cottage cheese (2–8%, 0.5 dL), and ice cream occasionally (~1150 mg calcium/day) C = habitual diet without changing the intake of dairy products (~625 mg calcium/day)	No
Zemel, 2004 [65]	I = High dairy diet (1200 to 1300 mg of dietary calcium/day supplemented with placebo). C = Standard dairy diet (400 to 500 mg of dietary calcium/day supplemented with placebo).	No
Zemel, 2005 [66] (Phase 1)	I = High dairy diet (1200 mg calcium/day including 3 servings/day of dairy with at least one in the form of fluid milk). C = Low dairy diet (500 mg/day calcium with <1 serving/day).	No
Zemel, 2005 [66] (Phase 2)	I = High dairy diet (1200 mg calcium/day including 3 servings/day of dairy with at least one in the form of fluid milk) with 500-kcal/d deficit. C = 0 to 1 servings of low-fat dairy products per day, and containing a total of <500 mg calcium per day with 500-kcal/day deficit.	No
Zemel, 2005 [67]	I = Yogurt diet providing a 500 kcal/day deficit and containing three daily six-ounce servings of a commercial fat-free yogurt (Yoplait Light), to bring the total calcium intake from 500­–1100 mg/day. C = 0 to 1 servings of dairy products/day and 400–500 mg calcium per day. The control diet incorporated as a placebo three daily servings of a sugar-free, calcium-free, prepackaged flavored gelatin dessert containing 10 kcal/serving with 500 kcal/day deficit.	No
Zemel, 2008 [68]	I = Recommended dairy diet (>3 servings/day of milk, cheese or yogurt). C = Low dairy diet (<1 dairy serving/day).	Yes
Zemel, 2009 [69]	I = High dairy diet (placebo supplemented) containing three daily servings dairy products (milk, cheese, and/or yogurt) substituted for other protein sources in the diet, to bring the total calcium intake to 1400 mg/day with a 500 calories/day deficit. C = 0 to 1 servings of dairy products/day, 500 mg calcium/day, and a daily placebo (methyl-cellulose) supplement with a 500 calories/day deficit.	No

Abbreviations: C, control group; DASH, dietary approaches to stop hypertension; I, intervention group; LDL-C, low density lipoprotein cholesterol; LFM, low-fat milk.

## Supplementary Materials

The following are available online at https://www.mdpi.com/2072-6643/11/9/2237/s1, Table S1: Randomized Controlled Trials of Dairy Intake not included in the Meta-Analysis, Figure S1: Funnel plot of All Studies Evaluating Dairy intake and Body Weight, Figure S2: Funnel plot of All Studies Evaluating Dairy intake and Waist Circumference, Figure S3: Funnel plot of All Studies Evaluating Dairy intake and HOMA-IR, Figure S4: Forest Plot of Randomized Clinical Trials with Low Risk of Bias: Dairy Intake and HOMA-IR, Figure S5: Forest Plot of Randomized Clinical Trials with Low Risk of Bias: Dairy Intake and Waist Circumference, Figure S6: Forest Plot of Randomized Clinical Trials with Low Risk of Bias: Dairy Intake and Body Weight, Figure S7: Forest Plot of Randomized Clinical Trials without Physical Activity Component: Dairy Intake and HOMA-IR, Figure S8: Forest Plot of Randomized Clinical Trials without Physical Activity Component: Dairy Intake and Waist Circumference, Figure S9: Forest Plot of Randomized Clinical Trials without Physical Activity Component: Dairy Intake and Body Weight.
