# Supplementary material for: The Effects of Dairy Intake on Insulin Resistance: A Systematic Review and Meta-Analysis of Randomized Clinical Trials
Source: Nutrients. 2019 Sep 17;11(9):2237. doi: 10.3390/nu11092237 (PMC6769921; doi:10.3390/nu11092237)
Supplement: Supplementary file 1 [file nutrients-11-02237-s001.pdf]

SUPPLEMENT
TABLE AND FIGURES

Table S1. Randomized Controlled Trials of Dairy Intake not included in the Meta-Analysis

Study Author, Year	Sample size	Sex	Age mean± SD or range (years)	BMI mean± SD or range (kg/m^2)	Country of origin	Study duration (weeks)	Findings<br><br>Intervention vs. Control
Agrawal, 2011 [23]	I=12<br>C=12	71%M<br>29%F	16.7±8.9	I=15.8±2.4<br>C=15.8±3.0	India	108	↓ BMI<br>↔ Insulin
Anderson, 2005 [16]	I=39<br>C=51	12%M<br>88%F	I=47.6±9.4<br>C=47.3±9.4	27-40	United States	12	↔ Body Weight<br>↔ WC
Alonso, 2009* [33]	I=23<br>C=22	51% M<br>49% F	I= 19.9±1.4<br>C=19.9±1.6	I=23.4±3.5<br>C=23.7±3.7	Spain	20	↑ Body Weight
Al Naggar, 2014	I=15<br>C=15	**	18-33	***	Malaysia	2	↓ Body Weight
Azadbakht, 2005 [21]	I=38<br>C=40	30%M<br>70%F	41.2±12.3	***	Iran	28	↓ Body Weight<br>↓ WC
Barr, 2000 [22]	I=98<br>C=102	36%M/<br>64%F	I=65.1±6.7C=<br>65.3±6.6	16-36	Canada	12	↑ Body Weight
Buchowski, 2010 [24]	I=17<br>C=17	29%M<br>71%F	21-50	29-35	United States	12	↔ Body Weight
Chee, 2003 [18]	I=91<br>C=82	100%F	I=58.7±3.7<br>C=59±3.2	23.8±3.6	Malaysia	108	↔ Body Weight<br>↔ %Body Fat<br>↔ Lean Mass
Dugan, 2014 [25]	I=14<br>C=23	36%M<br>64%F	54±9.7	94.4±19.9	United States	16	↔ Lean Mass<br>↓ Body Weight<br>↓ BMI<br>↓ WC
Eagan, 2006 [32]	I1=14<br>I2=14<br>C=10	100%M	20.1±2.4	I1=21.9±3.4<br>I2=23.4±4.7<br>C=21.9±2.6	United States	78	↓ Fat Mass
Lau, 2001 [17]	I=95<br>C=90	100%F	I=57.1±1.8<br>C=56.8±1.5	***	China	108	↑Body Weight
Liu, 2011 [26]	I=12<br>C=12	100%F	24-31	21-24	China	29	****
Maki, 2015 [27]	I=14<br>C=20	50%M<br>50%F	18-74	≤45	United States	14	↑ Insulin<br>↔ Body Weight<br>↔ WC<br>↑ HOMA-IR

Palacios, 2011 [28]	I=8 C=8	20%M 80%F	22-50	≥30	Puerto Rico	21	↔ Body Weight ↔ BMI ↔ Lean Mass ↔ Fat Mass ↔ %Body Fat
Rideout, 2013 [29]	I=23 C=23	20%M 80%F	18-75	18.5-35.0	Canada	52	↓ Insulin ↓ HOMA-IR ↔ Body Weight
Sirtori, 1999* [19]	21	38%M 62%F	51.9±13.5	***	Italy	4	****
Van Mejil, 2010 [30]	I=17 C=18	28%M 72%F	18-70	≥27	Netherlands	8	↔ Body Weight
Wagner, 2007 [20]	I=17 C=13	100%F	I= 37.6± 2.2 C=36.0± 2.2	I= 33.7±1.0 C=32.4±1.5	United States	12	↔ Body Weight ↔ Fat Mass
Zemel, 2010* [34]	I=10 C=10	70%M 30% F	31.0±10.3	28.0±1.01	United States	12	↔ Body Weight

*Cross-over design; **Data missing on sex; ***Data missing on BMI; ****Data missing on the outcome of body weight, waist circumference, or HOMA-IR. ↓= decrease; ↑ = increase; ↔ = no difference. Abbreviations: BMI, body mass index; C, control; F, female; HOMA-IR, Homeostatic Model Assessment of Insulin Resistance; I, intervention; M, male; SD, standard deviation; WC, waist circumference

Figure S1. Funnel plot of All Studies Evaluating Dairy intake and Body Weight

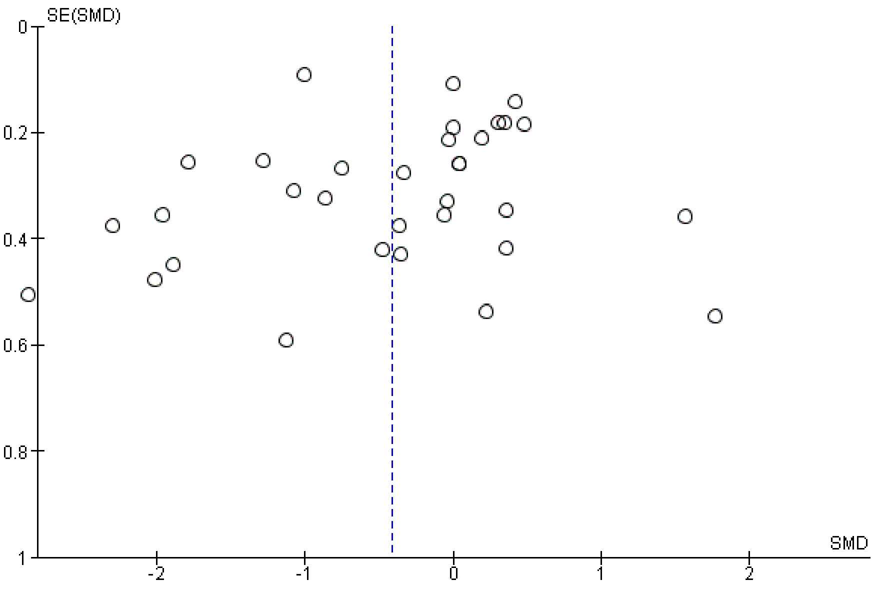

Figure S2. Funnel plot of All Studies Evaluating Dairy intake and Waist Circumference

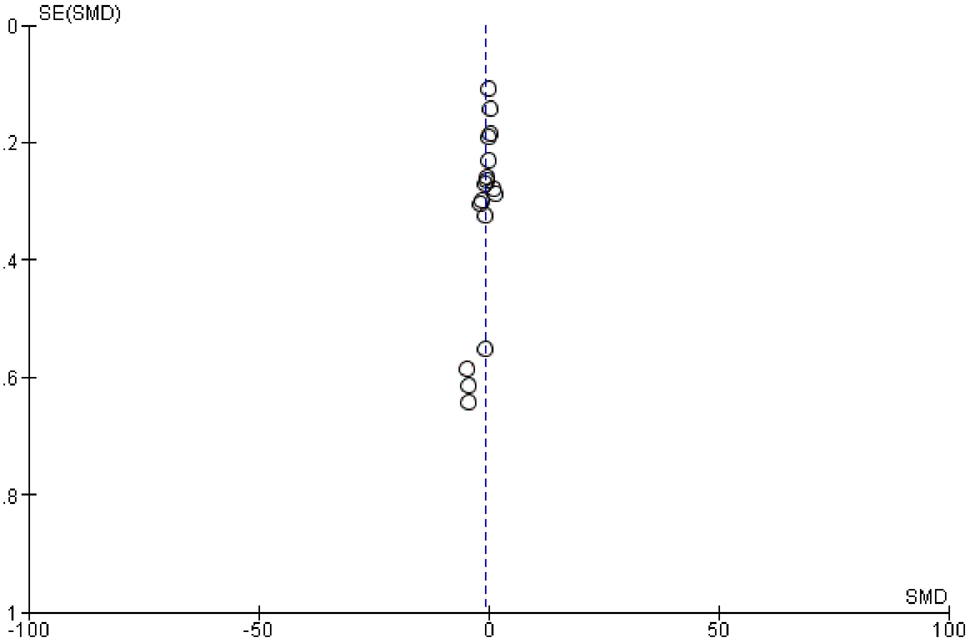

Figure S3. Funnel plot of All Studies Evaluating Dairy intake and HOMA-IR

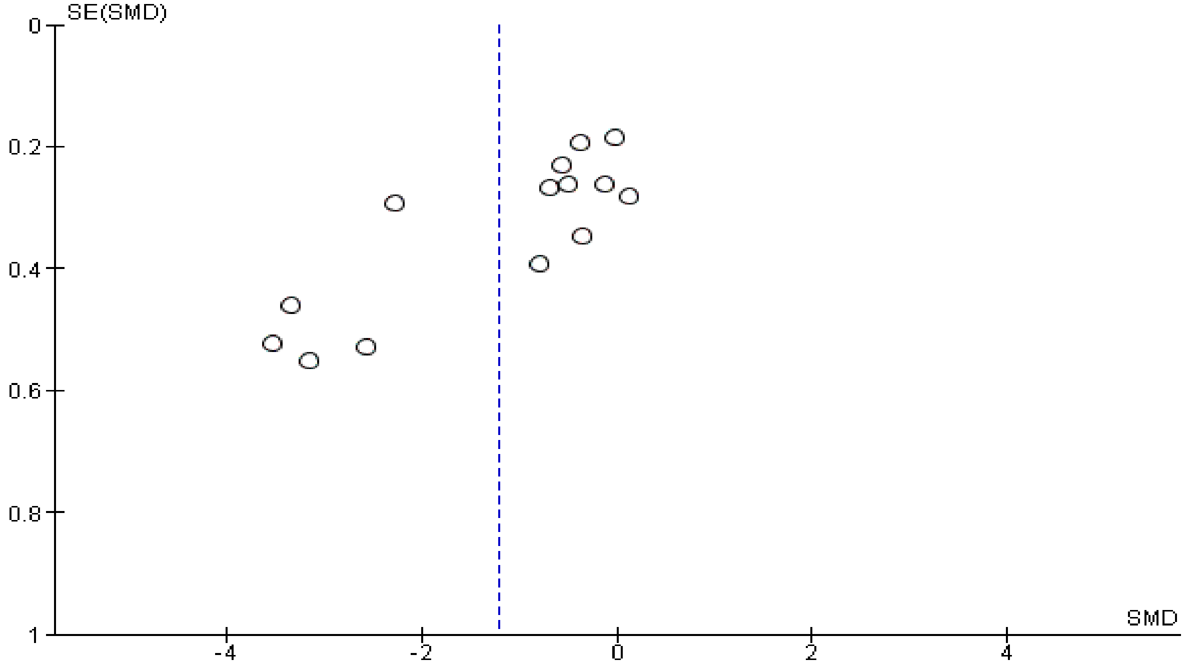

Figure S4. Forest Plot of Randomized Clinical Trials with Low Risk of Bias: Dairy Intake and HOMA-IR

	intervention			Control				Std. Mean Difference
**Study or Subgroup**	**Mean**	**SD**	**Total**	**Mean**	**SD**	**Total**	**Weight**	**IV, Random, 95% CI**
Adamsson 2011	-0.11	0.51	38	0.22	0.64	40	10.7%	-0.56 [-1.02, -0.11]
Adamsson 2015	-0.12	0.13	38	0.27	0.2	40	10.3%	-2.28 [-2.85, -1.70]
Asemi 2013	-0.8	0.43	16	1.1	0.71	16	8.2%	-3.16 [-4.23, -2.08]
Asemi 2015	-0.46	0.37	24	0.8	0.37	24	9.0%	-3.35 [-4.25, -2.45]
Razavi 2016	-0.8	0.8	30	-0.2	0.9	30	10.5%	-0.70 [-1.22, -0.17]
Thompson 2005 I1-HD	-1.27	1.59	31	-0.612	0.895	29	10.5%	-0.50 [-1.01, 0.02]
Thompson 2005 I2-HD+F	-0.717	0.688	30	-0.612	0.895	29	10.5%	-0.13 [-0.64, 0.38]
Wennersberg 2009	-0.6	6.2	55	3.1	12.2	54	10.9%	-0.38 [-0.76, -0.00]
Zemel 2005 phase 1	-0.72	0.44	17	-0.53	0.57	17	9.9%	-0.36 [-1.04, 0.31]
Zemel 2005 phase 2	-0.93	0.56	17	-0.57	0.18	12	9.5%	-0.78 [-1.55, -0.01]
**Total (95% CI)**			**296**			**291**	**100.0%**	**-1.14 [-1.72, -0.57]**

Heterogeneity: Tau² = 0.75; Chi² = 89.86, df = 9 (P < 0.00001); I² = 90%
Test for overall effect: Z = 3.89 (P < 0.0001)

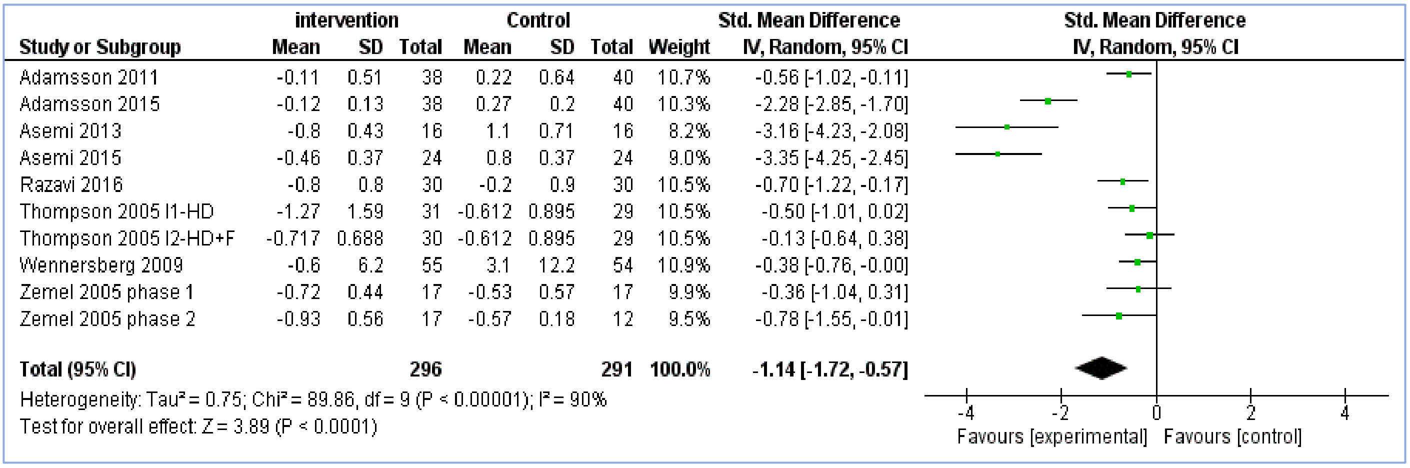

Abbreviations: F, fiber; HD, high dairy diet

Figure S5. Forest Plot of Randomized Clinical Trials with Low Risk of Bias: Dairy Intake and Waist Circumference

Study or Subgroup	intervention Mean	intervention SD	intervention Total	Control Mean	Control SD	Control Total	Weight	Std. Mean Difference IV, Random, 95% CI
Asemi 2015	-5.3	0.7	24	-1.9	0.7	24	7.4%	-4.78 [-5.92, -3.63]
Faghih 2011	-6.32	2.57	22	-3.98	2.77	20	9.1%	-0.86 [-1.50, -0.23]
Josse 2011 I1-MD	-1.8	0.7	30	-0.6	0.8	30	9.3%	-1.58 [-2.16, -0.99]
Josse 2011 I2-HD	-1.9	0.7	30	-0.6	0.8	30	9.2%	-1.71 [-2.30, -1.11]
Razavi 2016	-4.2	1.9	30	-2.6	1.7	30	9.4%	-0.88 [-1.41, -0.34]
Tanaka 2014	-1.5	4.1	102	-3.2	4.1	98	10.0%	0.41 [0.13, 0.69]
Thompson 2005 I1-HD	-11.6	6.1	30	-8.8	6.3	29	9.4%	-0.45 [-0.96, 0.07]
Thompson 2005 I2-HD+F	-11	7.6	31	-8.8	6.3	29	9.5%	-0.31 [-0.82, 0.20]
Wennersberg 2009	-0.9	3.1	55	-0.6	2.6	54	9.8%	-0.10 [-0.48, 0.27]
Zemel 2005	-3.99	0.48	18	-0.58	1.04	16	7.0%	-4.20 [-5.45, -2.94]
Zemel 2008	-7.5	4.3	169	-7.9	3.3	169	10.0%	0.10 [-0.11, 0.32]
**Total (95% CI)**			**541**			**529**	**100.0%**	**-1.14 [-1.73, -0.55]**

Heterogeneity: Tau² = 0.89; Chi² = 182.51, df = 10 (P < 0.00001); I² = 95%
Test for overall effect: Z = 3.78 (P = 0.0002)

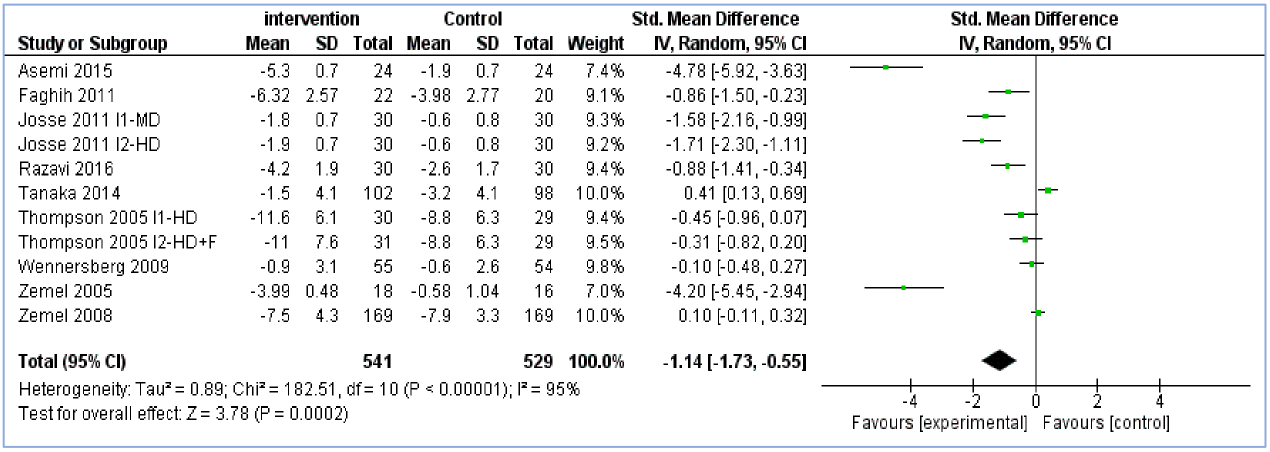

Waist circumference change measured in cm.
Abbreviations: F, fiber; HD, high dairy diet; MD, moderate dairy diet

Figure S6. Forest Plot of Randomized Clinical Trials with Low Risk of Bias: Dairy Intake and Body Weight

Study or Subgroup	intervention Mean	intervention SD	intervention Total	Control Mean	Control SD	Control Total	Weight	Std. Mean Difference IV, Random, 95% CI
Adamsson 2011	-3	1.86	44	0.03	1.47	42	4.5%	-1.79 [-2.29, -1.28]
Adamsson 2015	0.28	0.23	38	0.62	0.29	38	4.5%	-1.29 [-1.78, -0.79]
Appel 2003	-5.8	5.8	269	-1.1	3.2	273	4.9%	-1.00 [-1.18, -0.83]
Asemi 2015	-3.6	1.2	24	-1.3	1.1	24	4.1%	-1.97 [-2.66, -1.27]
Bendsen 2008	-1	0.7	11	-0.7	0.9	11	3.8%	-0.36 [-1.20, 0.49]
Faghih 2011	-4.43	1.93	22	-2.87	1.55	20	4.2%	-0.87 [-1.51, -0.23]
Farnsworth 2003(Females)	-6.6	0.5	21	-7.4	0.5	21	4.1%	1.57 [0.87, 2.27]
Farnsworth 2003(Males)	-11.9	2.1	7	-9.6	1.7	7	3.1%	-1.13 [-2.28, 0.03]
Gunther 2005 I1-HD	1.5	4.1	48	0.8	2.8	42	4.7%	0.20 [-0.22, 0.61]
Gunther 2005 I2-MD	0.7	3	45	0.8	2.8	42	4.6%	-0.03 [-0.45, 0.39]
Harvey-Berino 2005	-7.8	7	25	-5.5	6.7	29	4.4%	-0.33 [-0.87, 0.21]
Liu 2010 I1-MP	-0.11	1.55	60	-0.6	1.64	60	4.7%	0.31 [-0.05, 0.67]
Liu 2010 I2-MP+Iso	-0.03	1.67	60	-0.6	1.64	60	4.7%	0.34 [-0.02, 0.70]
Razavi 2016	-3.8	2.2	30	-2.3	1.7	30	4.5%	-0.75 [-1.28, -0.23]
Tanaka 2014	-1.1	3.3	102	-2.6	3.8	98	4.8%	0.42 [0.14, 0.70]
Thomas 2011	-2.6	4.5	15	-1.2	2.5	14	4.0%	-0.37 [-1.11, 0.37]
Thompson 2005 I1-HD	-8.8	7.5	30	-9.1	7	29	4.5%	0.04 [-0.47, 0.55]
Thompson 2005 I2-HD+F	-8.8	7.9	31	-9.1	7	29	4.5%	0.04 [-0.47, 0.55]
Wennersberg 2009	-0.1	2.5	55	-0.1	2.6	54	4.7%	0.00 [-0.38, 0.38]
Zemel 2005	-6.63	0.6	18	-4.99	0.5	16	3.5%	-2.88 [-3.88, -1.89]
Zemel 2005 phase 1	0.4	0.6	17	0.2	0.5	17	4.1%	0.35 [-0.32, 1.03]
Zemel 2008	-11.2	3.8	169	-11.2	3.3	169	4.9%	0.00 [-0.21, 0.21]
Zemel 2009	-4.61	0.63	23	-3.15	0.62	26	4.0%	-2.30 [-3.03, -1.56]
**Total (95% CI)**			**1164**			**1151**	**100.0%**	**-0.47 [-0.80, -0.14]**

Heterogeneity: Tau² = 0.57; Chi² = 291.40, df = 22 (P < 0.00001); I² = 92%
Test for overall effect: Z = 2.78 (P = 0.005)

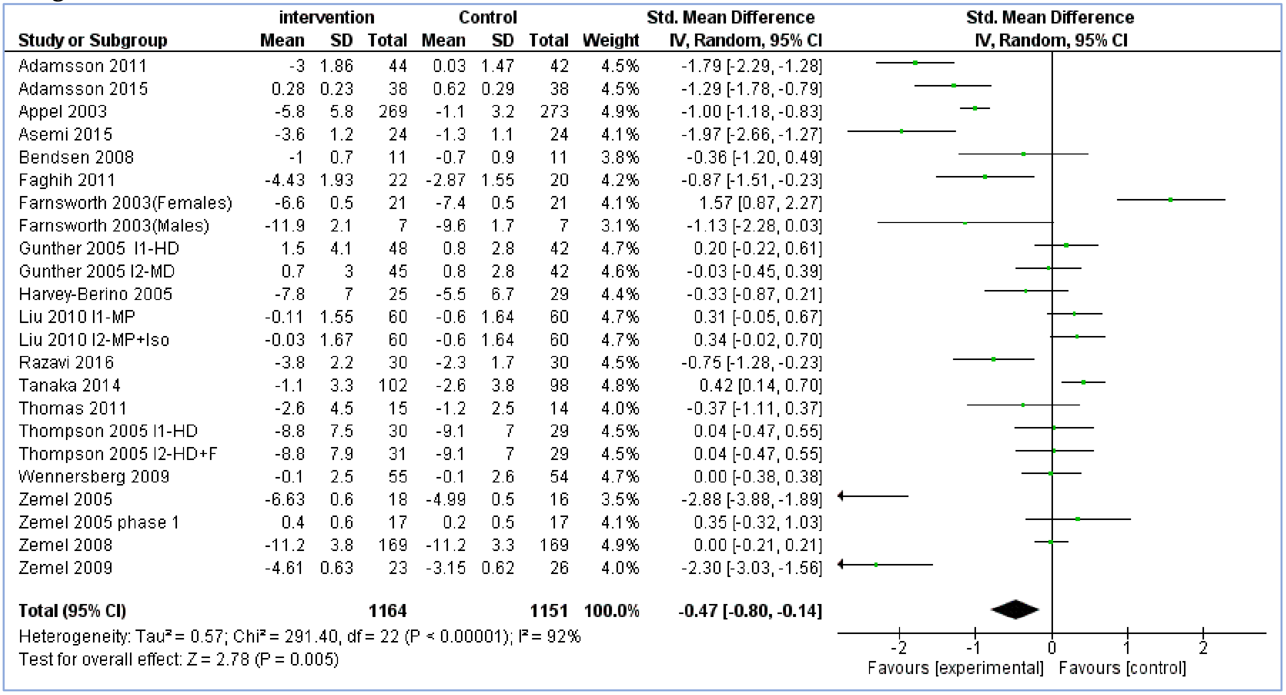

Body weight change measured in Kg.
Abbreviations: F, fiber; Iso, isoflavones; HD, high dairy diet; MD, moderate dairy diet; MP, milk protein

Figure S7. Forest Plot of Randomized Clinical Trials without Physical Activity Component: Dairy Intake and HOMA-IR

Study or Subgroup	intervention Mean	SD	Total	Control Mean	SD	Total	Weight	Std. Mean Difference IV, Random, 95% CI
Adamsson 2011	-0.11	0.51	38	0.22	0.64	40	8.9%	-0.56 [-1.02, -0.11]
Adamsson 2015	-0.12	0.13	38	0.27	0.2	40	8.7%	-2.28 [-2.85, -1.70]
Asemi 2013	-0.8	0.43	16	1.1	0.71	16	7.3%	-3.16 [-4.23, -2.08]
Asemi 2015	-0.46	0.37	24	0.8	0.37	24	7.9%	-3.35 [-4.25, -2.45]
Benatar 2014	0.08	0.99	58	0.09	0.69	60	9.1%	-0.01 [-0.37, 0.35]
Bowen 2005	-0.35	1.35	25	-0.615	2.87	25	8.7%	0.12 [-0.44, 0.67]
Razavi 2016	-0.8	0.8	30	-0.2	0.9	30	8.8%	-0.70 [-1.22, -0.17]
Stancliffe 2011	-0.59	0.34	20	1.19	0.61	20	7.5%	-3.53 [-4.56, -2.51]
Wennersberg 2009	-0.6	6.2	55	3.1	12.2	54	9.0%	-0.38 [-0.76, -0.00]
Zemel 2004	-1.22	0.51	14	-0.13	0.28	14	7.5%	-2.57 [-3.61, -1.53]
Zemel 2005 phase 1	-0.72	0.44	17	-0.53	0.57	17	8.4%	-0.36 [-1.04, 0.31]
Zemel 2005 phase 2	-0.93	0.56	17	-0.57	0.18	12	8.2%	-0.78 [-1.55, -0.01]
**Total (95% CI)**			**352**			**352**	**100.0%**	**-1.38 [-2.01, -0.75]**

Heterogeneity: Tau² = 1.11; Chi² = 149.30, df = 11 (P < 0.00001); I² = 93%
Test for overall effect: Z = 4.29 (P < 0.0001)

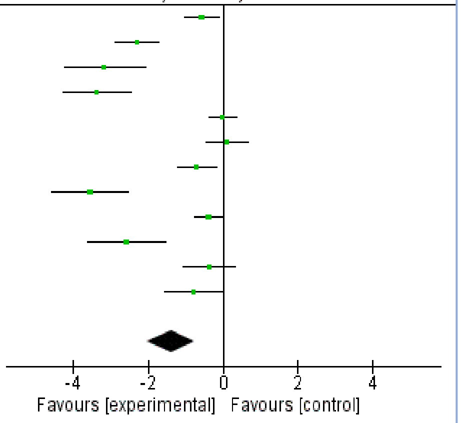

Figure S8. Forest Plot of Randomized Clinical Trials without Physical Activity Component: Dairy Intake and Waist Circumference

Study or Subgroup	intervention Mean	SD	Total	Control Mean	SD	Total	Weight	Std. Mean Difference IV, Random, 95% CI
Asemi 2015	-5.3	0.7	24	-1.9	0.7	24	10.2%	-4.78 [-5.92, -3.63]
Benatar 2014	0.4	3.1	58	-0.7	2.4	60	12.0%	0.40 [0.03, 0.76]
Faghih 2011	-6.32	2.57	22	-3.98	2.77	20	11.6%	-0.86 [-1.50, -0.23]
Lukaszuk 2007	-11.28	5.23	7	-8.66	2.51	7	10.4%	-0.60 [-1.68, 0.48]
Razavi 2016	-4.2	1.9	30	-2.6	1.7	30	11.8%	-0.88 [-1.41, -0.34]
Stancliffe 2011	-2.8	0.8	20	-0.2	0.1	20	10.0%	-4.47 [-5.67, -3.27]
Tanaka 2014	-1.5	4.1	102	-3.2	4.1	98	12.1%	0.41 [0.13, 0.69]
Wennersberg 2009	-0.9	3.1	55	-0.6	2.6	54	12.0%	-0.10 [-0.48, 0.27]
Zemel 2005	-3.99	0.48	18	-0.58	1.04	16	9.9%	-4.20 [-5.45, -2.94]
**Total (95% CI)**			**336**			**329**	**100.0%**	**-1.53 [-2.41, -0.64]**

Heterogeneity: Tau² = 1.66; Chi² = 188.18, df = 8 (P < 0.00001); I² = 96%
Test for overall effect: Z = 3.38 (P = 0.0007)

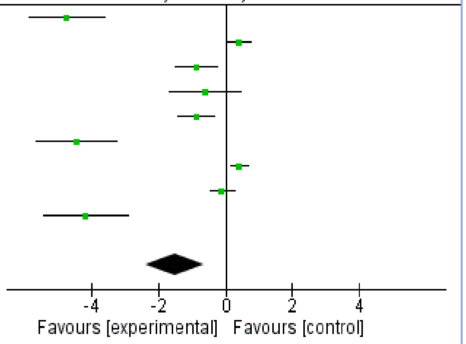

Waist circumference change measured in cm.

Figure S9. Forest Plot of Randomized Clinical Trials without Physical Activity Component: Dairy Intake and Body Weight

Study or Subgroup	intervention Mean	intervention SD	intervention Total	Control Mean	Control SD	Control Total	Weight	Std. Mean Difference IV, Random, 95% CI
Adamsson 2011	-3	1.86	44	0.03	1.47	42	4.8%	-1.79 [-2.29, -1.28]
Adamsson 2015	0.28	0.23	38	0.62	0.29	38	4.8%	-1.29 [-1.78, -0.79]
Asemi 2015	-3.6	1.2	24	-1.3	1.1	24	4.5%	-1.97 [-2.66, -1.27]
Benatar 2014	0.4	1.3	58	-0.2	1.2	60	5.0%	0.48 [0.11, 0.84]
Bendsen 2008	-1	0.7	11	-0.7	0.9	11	4.3%	-0.36 [-1.20, 0.49]
Bowen 2005(Females)	-9.4	1	15	-7.8	0.6	15	4.2%	-1.89 [-2.77, -1.01]
Bowen 2005(Males)	-9.4	1.3	10	-12	1.5	10	3.8%	1.77 [0.70, 2.84]
Faghih 2011	-4.43	1.93	22	-2.87	1.55	20	4.6%	-0.87 [-1.51, -0.23]
Farnsworth 2003(Females)	-6.6	0.5	21	-7.4	0.5	21	4.5%	1.57 [0.87, 2.27]
Farnsworth 2003(Males)	-11.9	2.1	7	-9.6	1.7	7	3.7%	-1.13 [-2.28, 0.03]
Gunther 2005 I1-HD	1.5	4.1	48	0.8	2.8	42	4.9%	0.20 [-0.22, 0.61]
Gunther 2005 I2-MD	0.7	3	45	0.8	2.8	42	4.9%	-0.03 [-0.45, 0.39]
Liu 2010 I1-MP	-0.11	1.55	60	-0.6	1.64	60	5.0%	0.31 [-0.05, 0.67]
Liu 2010 I2-MP+Iso	-0.03	1.67	60	-0.6	1.64	60	5.0%	0.34 [-0.02, 0.70]
Lukaszuk 2007	-3.76	2.25	7	-4.27	2.05	7	3.9%	0.22 [-0.83, 1.27]
Razavi 2016	-3.8	2.2	30	-2.3	1.7	30	4.8%	-0.75 [-1.28, -0.23]
Tanaka 2014	-1.1	3.3	102	-2.6	3.8	98	5.1%	0.42 [0.14, 0.70]
Wennersberg 2009	-0.1	2.5	55	-0.1	2.6	54	5.0%	0.00 [-0.38, 0.38]
Zemel 2004	-11.07	1.63	14	-6.6	2.58	14	4.1%	-2.01 [-2.94, -1.08]
Zemel 2005	-6.63	0.6	18	-4.99	0.5	16	4.0%	-2.88 [-3.88, -1.89]
Zemel 2005 phase 1	0.4	0.6	17	0.2	0.5	17	4.6%	0.35 [-0.32, 1.03]
Zemel 2009	-4.61	0.63	23	-3.15	0.62	26	4.5%	-2.30 [-3.03, -1.56]
**Total (95% CI)**			**729**			**714**	**100.0%**	**-0.50 [-0.91, -0.09]**

Heterogeneity: Tau² = 0.85; Chi² = 273.97, df = 21 (P < 0.00001); I² = 92%
Test for overall effect: Z = 2.38 (P = 0.02)

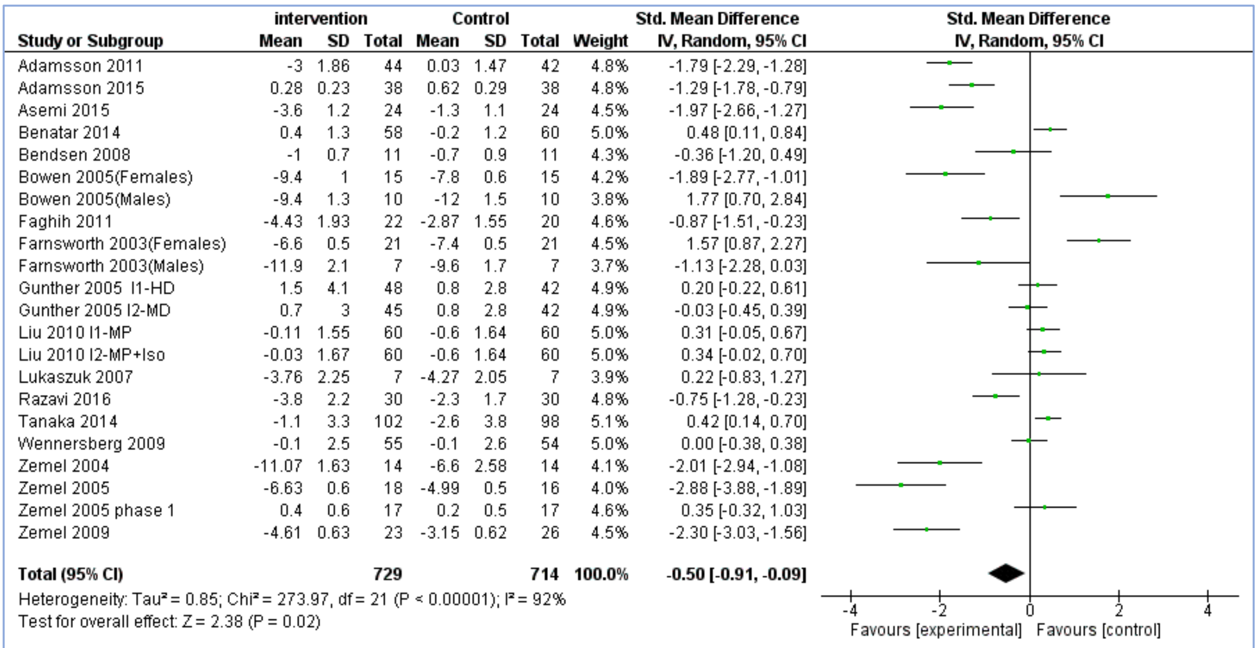

Body weight change measured in Kg.

Abbreviations: Iso, isoflavones; HD, high dairy diet; MD, moderate dairy diet; MP, milk protein
